# ZIF‐Co_3_O_4_@ZIF‐Derived Urchin‐Like Hierarchically Porous Carbon as Efficient Bifunctional Oxygen Electrocatalysts

**DOI:** 10.1002/open.202400057

**Published:** 2024-06-10

**Authors:** Lingling Zhang, Xia Wang, Chong Gong, Weiyan Sun, Zihan Lu

**Affiliations:** ^1^ Haidu college Qingdao Agriculture University Yantai 265200 China; ^2^ College of Materials Science and Engineering Qingdao University of Science and Technology Qingdao 266042 China

**Keywords:** ZIF, Layered porous carbon, electrocatalyst, ORR, OER

## Abstract

Co_3_O_4_ nanoparticles were sandwiched into interlayers between ZIF‐8 and ZIF‐67 to form ZIF‐Co_3_O_4_@ZIF precursors. Pyrolysis of ZIF‐Co_3_O_4_@ZIF yielded an urchin‐like hierarchically porous carbon (Co@CNT/NC), the thorns of which were carbon nanotubes embedded Co nanoparticles. With large specific surface area and hierarchically porous structure, as‐prepared Co@CNT/NC exhibited excellent bifunctional oxygen electrocatalytic performances. It has good ORR performance with E_1/2_ of 0.85 V, which exceeds the Pt/C half‐wave potential (E_1/2_=0.83 V). In addition, Co@CNT/NC has an OER performance close to that of RuO_2_. To further demonstrate the effect of Co modifying on the properties, the samples were subjected to acid washing treatment. Co‐based nanoparticles were proved to After acid washing, there was obvious loss of Co particles in Co@CNT/NC, resulting in poor oxygen electrocatalysis. So, the pyrolysis products of ZIF‐8‐Co_3_O_4_@ZIF‐67 retained large specific surface area and porous structure can be retained, and on the other hand, the carbon tube structure and original polyhedron framework. Besides, existence of Co nanoparticle@carbon nanotube provided more active sites and improved the ORR and OER performances.

## Graphical abstract

Pyrolysis of ZIF‐Co_3_O_4_@ZIF yielded an urchin‐like hierarchically porous carbon (Co@CNT/NC), the thorns of which are carbon nanotubes embedded Co nanoparticles. As‐prepared Co@CNT/NC exhibits excellent bifunctional oxygen electrocatalytic performances. To further demonstrate the effect of Co modifying on the properties, the samples are subjected to acid washing treatment. The active site of catalyst is further proved.

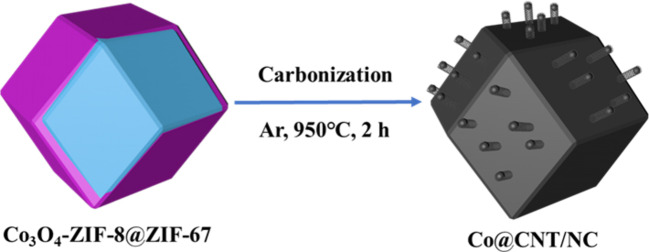

## 1.Introduction

In recent years, with the gradual depletion of traditional energy sources and growing concern for the environment, the demand for environmentally friendly renewable energy sources has been increasing, driving the development of sustainable energy conversion and subsequent energy storage technologies.[^1^, ^2^, ^3^] Rechargeable metal‐air batteries have great potential due to their high energy density, abundant raw materials, reliable and safe operation, and low environmental impact.^[4]^ Unfortunately, the poor kinetic properties of catalysts for the oxygen reduction reaction (ORR) and oxygen evolution reaction (OER), which are the key reactions in rechargeable metal‐air batteries, lead to poor rechargeability and high overpotential, severely hindering their further applications.[^5^, ^6^, ^7^, ^8^, ^9^] At the beginning of the research, nanoparticles based on the nanostructure of precious metals (Ru, Pt) received attention as an effective catalyst.^[10]^To date, noble metal‐based catalysts such as platinum (Pt) and iridium (Ir) /ruthenium (Ru) remain the most established and effective ORR and OER electrocatalysts, respectively.[^11^, ^12^, ^13^] However, the high cost, low stability, and single catalytic function of these noble metals in monolithic catalysts have hindered their widespread commercialization.^[14]^ Therefore, it is crucial to explore cost‐effective and high‐performance bifunctional oxygen electrocatalysts.

Carbon‐based materials with a wide range of sources, large specific surface area and good electrical conductivity have been of great interest, however their low intrinsic activity is a major challenge.[^15^, ^16^] The doping of transition metals (Fe, Co, Ni) and heteroatoms (N, P, S, B) and the compounding of transition metal oxides can significantly improve the catalytic activity of carbon‐based materials.^[17]^ On the one hand, pyridine nitrogen, graphitic nitrogen and M‐N_x_ bonding of transition metals with nitrogen atoms all provide new ORR active sites for carbon‐based catalysts.^[18]^ On the other hand, the composite of transition metal or transition metal oxide nanoparticles with thin layers of graphitic carbon not only facilitates the dispersion and stabilization of nanoparticles, but also provides new OER active sites for carbon‐based catalysts.[^6^, ^7^] In addition, the doping of atoms in graphitic carbon changes the original charge distribution and spin density, which reduces the adsorption potential of O_2_ and peroxide intermediates and improves the electrocatalytic activity of ORR and OER.[^19^, ^20^] However, during the doping process, the transition metal atoms are prone to carbonization, nitration, and self‐aggregation, leading to problems of reduced doping, inhomogeneous metal particle size, and poor dispersion.^[21]^

Carbon‐based materials have attracted the most attention due to their excellent mechanical strength, high chemical and thermal stability, and good adsorption capacity. In order to prepare highly efficient heterogeneous carbon‐based catalytic systems, nanoparticles have been decorated on various supporting materials and have been widely used.^[22]^ For example, Das et al. prepared catalysts based on reduced graphene oxide/silver nanoparticles that can degrade multiple contaminations.^[22]^

Herein, metal organic skeletons (MOFs) are a class of porous crystalline materials formed by metal ion clusters and organic ligand coordination with a periodic network structure that can be extended indefinitely. As precursor templates for new porous carbon materials, they can effectively prevent self‐aggregation and sintering problems of metals and heteroatoms during pyrolysis.[^23^, ^24^] The size and structural dependence of MOFs determine the catalytic activity, the structural chemistry of the active nanoparticles, the reconstruction of the nanoparticle surface, and the diffusion function that controls the catalytic activity.^[25]^ We can adjust the size of the MOFs to change the composition of the polymer to customize the surface charge.^[26]^ The core‐shell structure obtained by the composite customization of ZIF materials can also make up for the defect of single catalytic site of single ZIF to a certain extent. The presence of Zn atomic nodes in ZIF‐8 not only provides replacement sites for active transition metal atoms, but also volatilizes above 900 °C to remove the formation of micropores.[^27^, ^28^, ^29^] Under the protection of inert gas or reducing atmosphere, a small amount of Co atoms will form Co‐N_x_ active sites with the in situ modifying of the polymer by pyrolysis; a large amount of Co atoms will catalyze the formation and growth of carbon nanotubes in the form of metal clusters.[^30^, ^31^, ^32^] Therefore, this chapter is designed to load an appropriate amount of Co_3_O_4_ nanoparticles on the surface of ZIF‐8, which in turn wraps ZIF‐67 to form ZIF‐8‐Co_3_O_4_@ZIF‐67 precursor. This precursor was pyrolyzed at high temperature under argon atmosphere to form a polyhedral carbon material modified by nitrogen‐modified carbon nanotubes encapsulated with Co nanoparticles, which exhibited excellent ORR and OER properties.

## Experimental

### Preparation of Co_3_O_4_ Nanoparticles

The classical hydrothermal method was used for the synthesis. 0.5 g of Co(CH_3_COO)_2_ ⋅ 4H_2_O was dissolved in 25 mL of water, 2.5 mL of ammonia was added with stirring, and stirring was continued for 10 min, then transferred to an autoclave and heated at 150 °C for 3 h. After natural cooling to room temperature, the synthesized black pellets were washed by centrifugation with deionized water three times and dried under vacuum. Depending on the type of solvent used (water or ethanol) and the volume of solvent, the diameter and morphology of the resulting particles can be controlled. The diameter of the Co_3_O_4_ nanoparticles synthesized in this paper is about 20 nm, and the morphology is that of nanospheres.

### Preparation of Co@CNT/NC Catalysts

Synthesis of ZIF‐8: 2.35 g of Zn(NO_3_)_2_ ⋅ 6H_2_O was first dissolved in 60 mL of methanol, then 5 g of 2‐methylimidazole was dissolved in 20 mL of methanol, stirred well and poured into Zn(NO_3_)_2_ ⋅ 6H_2_O solution to make the two mixed well. After stirring the reaction for 8 h, the prepared white ZIF‐8 product was collected by centrifugation and washed twice with methanol. After vacuum drying at 60 °C overnight, the white ZIF‐8 was ground into a fine powder in preparation for use in the next step.

Synthesis of Co@CNT/NC nanocatalysts: The powder ZIF‐8 was dissolved in 60 mL of methanol and stirred vigorously with sonication to make ZIF‐8 uniformly distributed in the solution. After that, 1.18 g of Co(NO_3_)_2_ ⋅ 6H_2_O and 1.34 g of 2‐MIN were dissolved in 10 mL of methanol, and then Co(NO_3_)_2_ ⋅ 6H_2_O methanol solution, 50 mg of Co_3_O_4_ nanoparticles and 2‐MIN methanol solution were added sequentially, respectively. After stirring for 2 h, the solution was left to stand for 24 h to stabilize the structure by aging. The product Co_3_O_4_‐ZIF‐8@ZIF‐67 was obtained after centrifugation, washing and drying. then the product was pyrolyzed under Ar atmosphere at a heating rate of 5 °C min^−1^ for 2 h at 950 °C. The final product Co@CNT/NC was then obtained. for comparison also CoNC after the pyrolysis of Co_3_O_4_‐ZIF‐8@ZIF‐8 was synthesized for comparison to demonstrate the importance of the ZIF‐67 shell in the formation of carbon tubes. In addition, further acid washing and secondary pyrolysis of Co@CNT/NC were performed to demonstrate the critical role of metal particle modifying in the performance by performance testing.

### Electrochemical Measurements

Electrochemical tests were performed on a CHI 760E electrochemical workstation in a standard three‐electrode configuration with a rotating disc electrode (RDE, diameter: 3 mm). An Ag/AgCl electrode and a graphite rod were used as the reference electrode and counter electrode, respectively. The electrocatalytic test drip ink was prepared as follows: 4 mg of catalyst and 1 mg of conductive carbon black were dissolved in 800 μL of deionized water and 200 μL of isopropanol and sonicated to make a uniform dispersion of the electrocatalyst. Before testing, 30 μL of Nafion (5 wt %) was added to the ink, and 3 μL of catalyst ink was slowly added dropwise to the rotating disc electrode and baked dry using an infrared lamp. The catalyst loading on the electrode was 0.31 mg cm^−2^. Cyclic voltammetry (CV) tests were studied at a scan rate of 50 mV s^−1^ between −0.05 and 1.1 V (vs. RHE) in a 0.1 M KOH solution saturated with N_2_ and O_2_. Then, linear scanning voltammetry (LSV) curves were studied at a scan rate of 10 mV s^−1^ and at different rotational speeds (400‐2500 rpm). OER performance was measured by LSV in O_2_‐saturated KOH (0.1 M) solutions at a scan rate of 10 mV s^−1^.

## 1 Results and Discussion

**Scheme 1:**
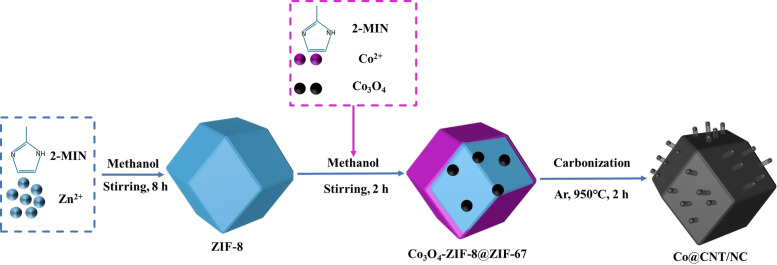
Schematic synthesis process of Co@CNT/NC.

A schematic diagram of the synthesis process of Co@CNT/NC is shown in Scheme 1. Firstly, Co_3_O_4_ nanoparticles were loaded onto ZIF‐8 in methanol. Then, Co(NO_3_)_2_ ⋅ 6H_2_O and 2‐MIM were added and stirred for 2 h. After aging 24 h, ZIF‐8‐Co_3_O_4_@ZIF‐67 precursors were collected and washed with water for three times. The dried product was then pyrolyzed at 950 °C under Ar atmosphere to obtain Co@CNT/NC.

**Figure 1:**
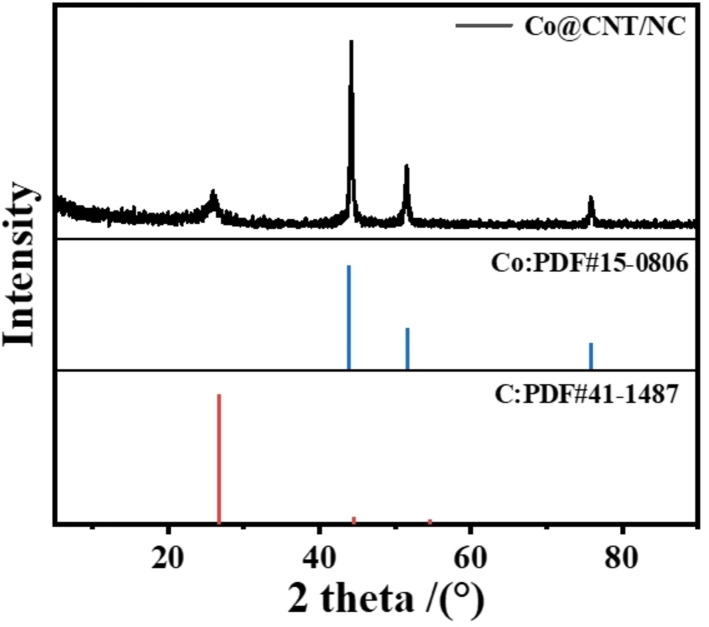
XRD patterns of Co@CNT/NC.

XRD patterns of Co@CNT/NC are shown in Figure 1. The broad peak shown around 26°, which corresponds to the (002) crystal plane of the graphite structure (PDF#41‐1487), indicates a good graphitization of the material, which provides a good electrical conductivity to the material. While the three peaks at around 44°, 51.5° and 76° match the (001), (004) and (110) crystal planes of the Co crystal phase in the material (PDF#15‐0806), indicating a good modifying of Co in the material. The XRD image of sample ZIF‐8‐Co_3_O_4_@ZIF‐67 is shown in Figure S1, when C is not yet graphitized. The diffraction peak corresponding to Co_3_O_4_ is relatively weak due to the small size of the nanoparticle.

**Figure 2:**
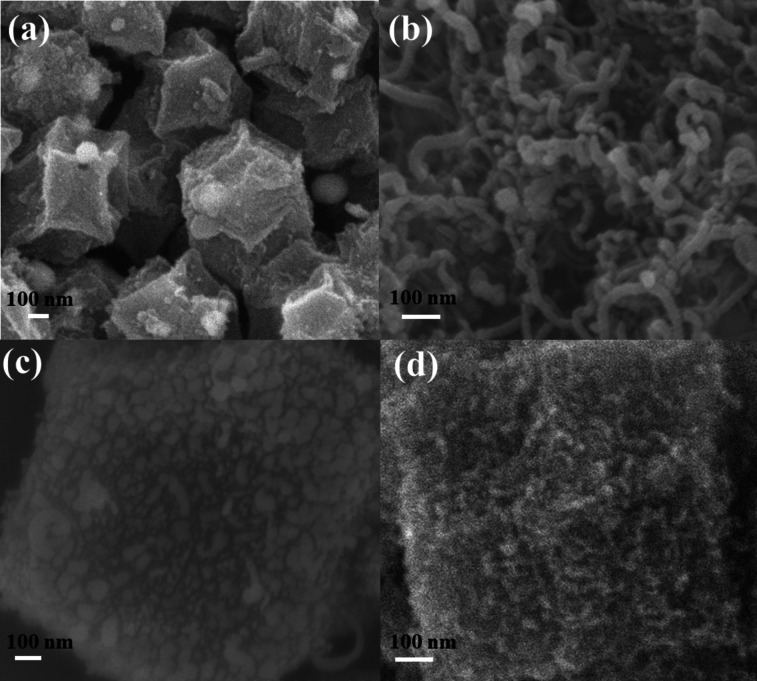
(a) SEM images of pyrolysis products of (a) ZIF‐8‐Co_3_O_4_, (b) ZIF‐67‐Co_3_O_4_, (c) ZIF‐8‐Co_3_O_4_@ZIF‐8 and (d) ZIF‐8‐Co_3_O_4_@ZIF‐67.

The morphological characteristics of the pyrolysis products of Co_3_O_4_‐ZIF‐8, Co_3_O_4_‐ZIF‐67, Co_3_O_4_‐ZIF‐8@ZIF‐8 and Co_3_O_4_‐ZIF‐8@ZIF‐67 can be observed from the SEM photographs in Figure 2. As can be seen in Figure 2 (a), the original dodecahedral structure of Co_3_O_4_‐ZIF‐8 was basically maintained after pyrolysis, and the surface was folded due to pyrolysis, and some Co_3_O_4_ nanoparticles of about 20 nm size were attached to the surface. In contrast, ZIF‐67 in Figure 2 (b), after high temperature pyrolysis, the original dodecahedral structure was destroyed, and it grew into a carbon nanotube structure covered with nanoparticles intertwined like water plants. Figure 2 (c) shows the Co/NC morphology of Co_3_O_4_‐ZIF‐8@ZIF‐8 synthesized as a comparison sample after pyrolysis, and the diameter of the product increases significantly because of the surface loading layer of ZIF‐8 to form a core‐shell structure. The surface‐loaded shells partially melted and destroyed during the pyrolysis, forming intermittent granular‐loaded shells. Figure 2 (d) shows the morphological characteristics of the final product Co@CNT/NC, which basically retains the original dodecahedral metal framework structure with large specific surface area and porous structure after high temperature carbonization at 950 °C. The size distribution for Co@CNT/NC is shown in Figure S3.On the other hand, the surface coating of a short and dense layer of carbon nanotubes with encapsulated metal nanoparticles can also greatly improve the catalytic performance. The SEM images and HADDF‐STEM images as a supplement are given in Figure S2 and Figure S4. The HAADF‐STEM images of Co@CNT/NC and the corresponding elemental mapping images (C, N, Co, Zn) are shown in Figure S4. The uniform distribution of C, N, and Co in the sample Co@CNT/NC proves that the elemental Co and nitrogen are well modified into the graphitic carbon substrate^[33]^. And the element Zn almost disappeared because under the pyrolysis at 950 °C, Zn was evaporated and produced micropores, which can be used as the carrier of active sites.

**Figure 3:**
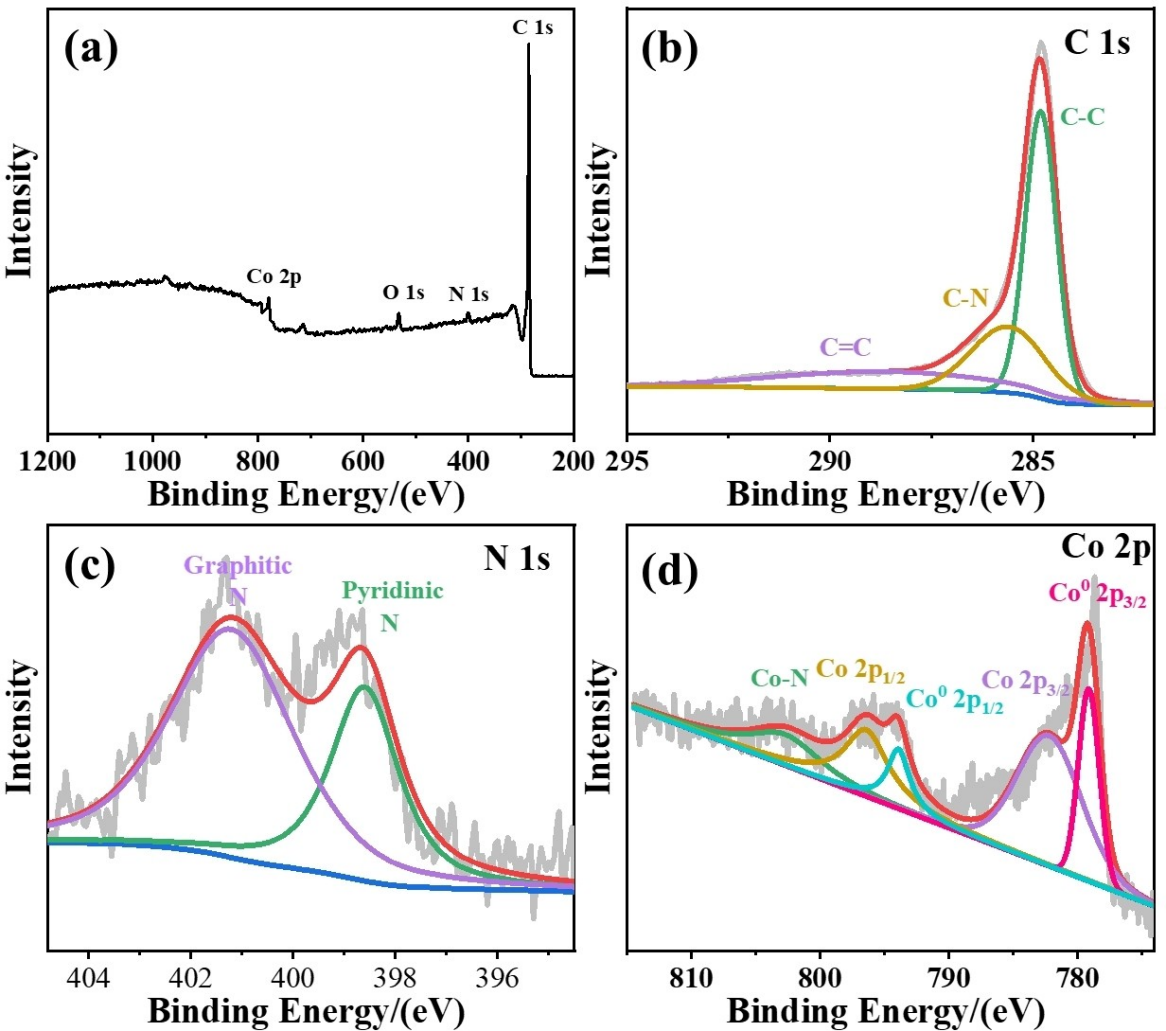
(a) The survey XPS spectrum of Co@CNT/NC catalyst. (b) The narrow spectra of C 1s in Co@CNT/NC catalyst. (c) The narrow spectra of N 1s in Co@CNT/NC catalyst. (d) The narrow spectra of Co 2p in Co@CNT/NC catalyst.

The chemical composition and electronic structure of Co@CNT/NC can be further characterized by XPS analysis. The total spectrum of Co@CNT/NC in Figure 3 (a) shows that elements containing C, N, O, and Co can be found in the XPS spectrum, while the signal of Zn is weak due to its low content in Co@CNT/NC due to the large amount of evaporation as mentioned above. From the fine spectra of C 1s (Figure 3 (b)), it can be seen that C 1s has three characteristic peaks with binding energies of 284.8 eV, 285.6 eV, and 288.6 eV, corresponding to C−C, C−N, and C=C chemical bonds, respectively. In contrast, the fine spectrum of N 1s (Figure 3 (c)) consists of only two broad peaks at 398.3 eV and 401.1 eV, which correspond to pyridine‐N and graphite‐N. Many literatures have reported that abundant pyridine‐N can induce charge deviation and coordination with metal atoms to form M‐N_x_ active sites, while graphite‐N facilitates the transport of intermediates and electron transfer during ORR and OER. Therefore, the high graphite‐N and pyridine‐N contents are the basis for the good performance of Co@CNT/NC materials. The fine spectra of Co 2p (Figure 3 (d)) can be decomposed into 779.1 eV, 781.6 eV, 793.9 eV, 796.4 eV and 802.7 eV, where the peaks of 781.6 eV and 796.4 eV originate from the higher valence states of Co 2p_3/2_and Co 2p_1/2_, demonstrating the partial residue of Co_3_O_4_ in Co@CNT/NC. While 779.1 eV and 793.9 eV correspond to Co^0^ 2p_3/2_ and Co^0^ 2p_1/2_ in the 0‐valent state, indicating the presence of Co nanoparticles resulting from the reduction of cobalt in Co_3_O_4_ and ZIF‐67 during pyrolysis, most of which are encapsulated in carbon tubes as metal cores for carbon tube growth.[^34^, ^35^] 802.7 eV peak corresponds to the Co@CNT/NC Co ‐N_x_ fraction, Co‐N_x_ is the better ORR and OER active site, indicating good modifying of Co and N in the carbon substrate, as also evidenced from the elemental mapping images.

**Figure 4:**
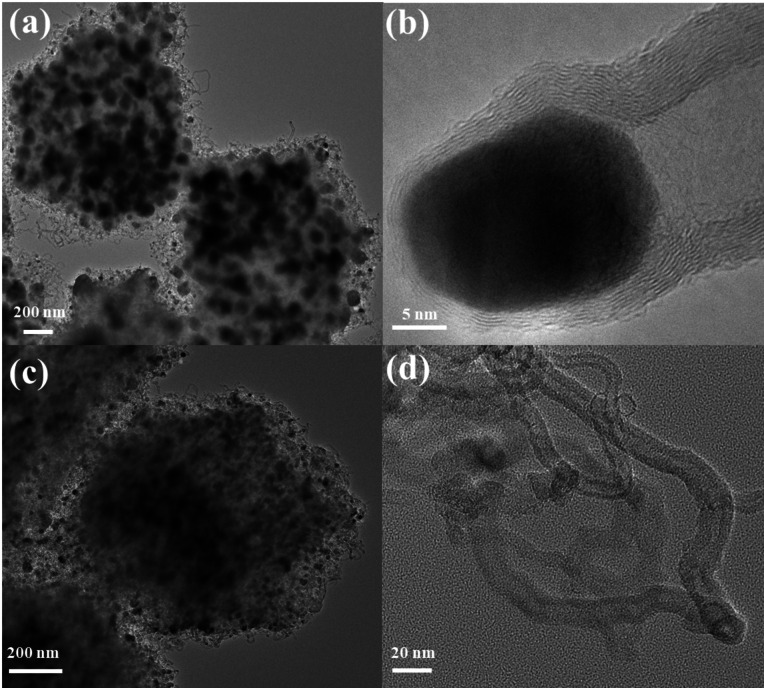
(a) TEM image of Co@CNT/NC. (b) HRTEM image of carbon nanotubes coated with metal nanoparticles. (c) TEM image of Co@CNT/NC after pickling. (d) HRTEM image of the acid‐washed carbon nanotubes.

The morphology and structure of Co@CNT/NC were further analyzed by TEM electron microscopy. As shown in Figure 4 (a), Co@CNT/NC keeps the dodecahedral shape better, and the surface is covered with a short and dense layer of carbon nanotubes covered with nanoparticles intertwined like water plants, which corresponds to the surface morphology observed by SEM photographs. In addition, there are many Co_3_O_4_ nanoparticles around 20 nm inside the Co@CNT/NC, and these metal oxide nanoparticles can also provide some catalytic properties to the catalyst. Figure 4 (b) further shows the morphology of the carbon nanotubes covered with the encapsulated nanoparticles. The carbon layer of the carbon nanotubes and the encapsulated nanoparticles can be clearly seen. The acid‐washed Co@CNT/NC is shown in Figure 4 (c). Compared with the photo before acid‐washing, the dodecahedral structure of Co@CNT/NC and the carbon tubes on the surface remain basically unchanged, but the metal nanoparticles of about 20 nm in the center are basically completely washed away. Further observation of the carbon tubes after acid washing (Figure 4 (d)) shows that the encapsulated metal nanoparticles in the carbon tubes are also basically washed away, leaving only empty carbon tubes.

In order to explore its porosity characteristics, N_2_ adsorption/desorption measurements were carried out on Co@CNT/NC and Co_3_O_4_‐ZIF‐8@ZIF‐8_950_, and the results are shown in Figure S5 (a). The type IV isotherm of the adsorption and desorption curves showed obvious hysteresis curves, indicating the mesoporous properties of the sample. Meanwhile, the rapid absorption and desorption of N_2_ in the range of low relative pressure indicated the presence of micropores. As shown in Figure S5 (b), the two samples have a similar number of mesopres, which is due to the fact that both of them are obtained by the combination of ZIF. In addition, Co@CNT/NC shows a higher specific surface area, which means more active sites can be exposed.

**Figure 5:**
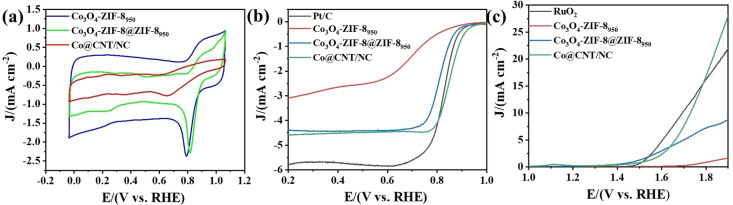
(a) CV curves with a sweeping rate of 50 mV s^−1^ in O_2_‐saturated 0.1 M KOH. (b) ORR polarization curves of Pt/C, NC, Co/NC and Co@CNT/NC in O_2_‐saturated 0.1 M KOH. (c) OER performance of Pt/C, NC, Co/NC and Co@CNT/NC with a scan rate of 10 mV s^−1^ at 1600 rpm.

The ORR and OER performance of Pt, NC, Co/NC and Co@CNT/NC under alkaline conditions are shown in Figure 5. Figure 5 (a) and Figure 5 (b) show the sample CV scan curves and LSV scan curves, respectively. Among the four samples, Co@CNT/NC has the best ORR half‐wave potential with E_1/2_ of 0.85 V, which exceeds the Pt/C half‐wave potential (E_1/2_=0.83 V). However, the limiting current density j_l_ of 4.58 mA cm^−2^ for Co@CNT/NC is lower than that of Pt (j_l_=5.777 mA cm^−2^), which is caused by the low intrinsic conductivity of the ZIFs metal framework after pyrolysis. Moreover, the E_1/2_ of NC and Co/NC were 0.68 V and 0.81 V, respectively, and j_l_ was 3.09 mA cm^−2^ and 4.39 mA cm^−2^, respectively. The results suggest that the modifying of Co and the carbon nanotube cladding of the encapsulated metal particles provide new active sites (Co‐N_x_, Co nanoparticles and Co_3_O_4_ oxides), which further improve the catalyst conductivity and catalytic performance. In addition, the LSV plot of OER (Figure 5 (c)), Co@CNT/NC has an overpotential E_j=10_=500 mV at a current density of 10 mA cm^−2^, which is slightly higher than RuO_2_ (464 mV) and better than NC and Co/NC. the OER results demonstrate that the modifying of Co and the encapsulation of carbon nanotubes with Co nanoparticles have a very significant effect on the improvement of OER performance effect. We compared its ORR and OER properties with other ZIF‐based composite nanostructures reported in the literature, as shown in Table S1.

**Figure 6:**
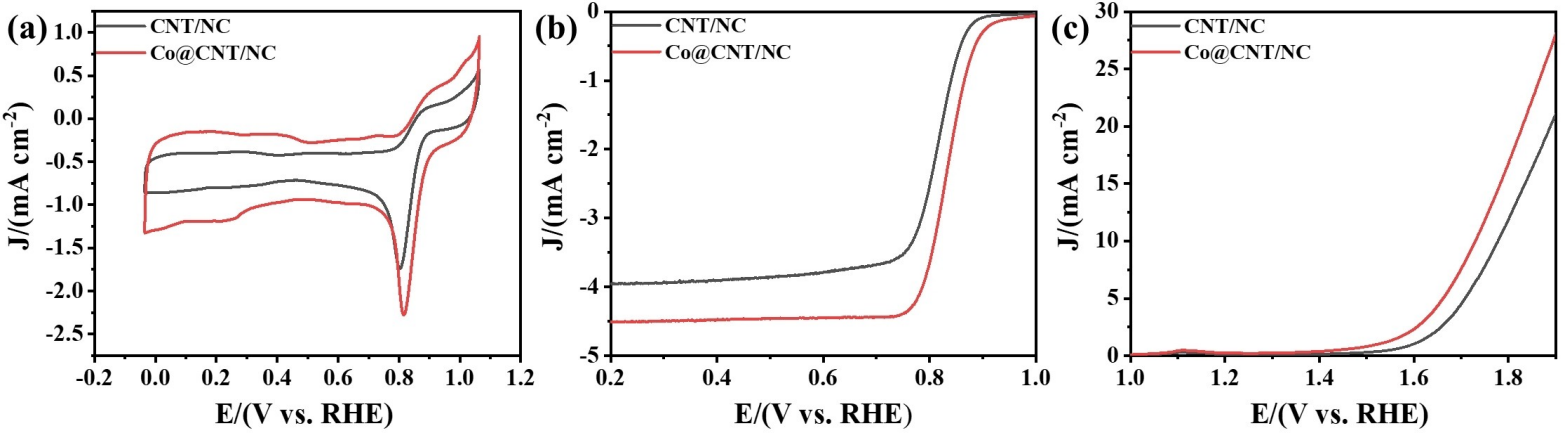
(a) CV curves of acid‐washed CNT/NC and Co@CNT/NC in O_2_‐saturated 0.1 M KOH with a scan rate of 50 mV s^−1^. (b) ORR polarization curves of acid‐washed CNT/NC and Co@CNT/NC in O_2_‐saturated 0.1 M KOH. (c) OER performance of the acid‐washed CNT/NC at 1600 rpm with a scan rate of 10 mV s^−1^.

To further demonstrate the role of Co nanoparticles and Co_3_O_4_ loading, acid washing and secondary pyrolysis were performed on Co@CNT/NC. After acid washing, the dodecahedral framework of the samples did not change much, but the Co nanoparticles encapsulated in carbon tubes and the modified Co_3_O_4_ were washed off (Figure 4 (c‐d)). The ORR and OER performance of the acid‐washed samples are shown in Figure 6 (a–c), and it can be seen that there is a relatively large decrease in the ORR performance and OER performance of the acid‐washed samples, which further proves the catalytic effect of Co_3_O_4_ oxide and Co nanoparticle loading.

**Figure 7:**
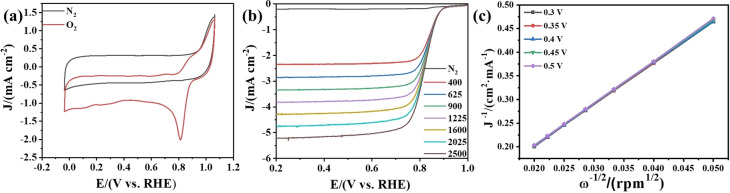
(a) CV curves of Co@CNT/NC at a scan rate of 50 mV s^−1^ in N_2_‐saturated and O_2_‐saturated 0.1 M KOH, respectively. (b) LSV curves of Co@CNT/NC in N_2_‐saturated and O_2_‐saturated 0.1 M KOH solutions at different rotational speeds. (c) K–L curve of Co@CNT/NC.

To further explore the reaction kinetics, the CV and LSV curves of the samples were tested in saturated N_2_ and O_2_ solutions at different rotation rates (Figure 7 (a–b)). The test under N_2_ was mainly used as a substrate to reduce the testing error. The K–L curves of the samples (Figure 7 (c)) can be calculated from the LSV curves at different rotation rates. The slope obtained by linear fitting can be calculated to obtain the electron transfer number n=3.8 for the sample, which indicates that the catalyst is mainly a four‐electron transfer process with good kinetic performance.

**Figure 8:**
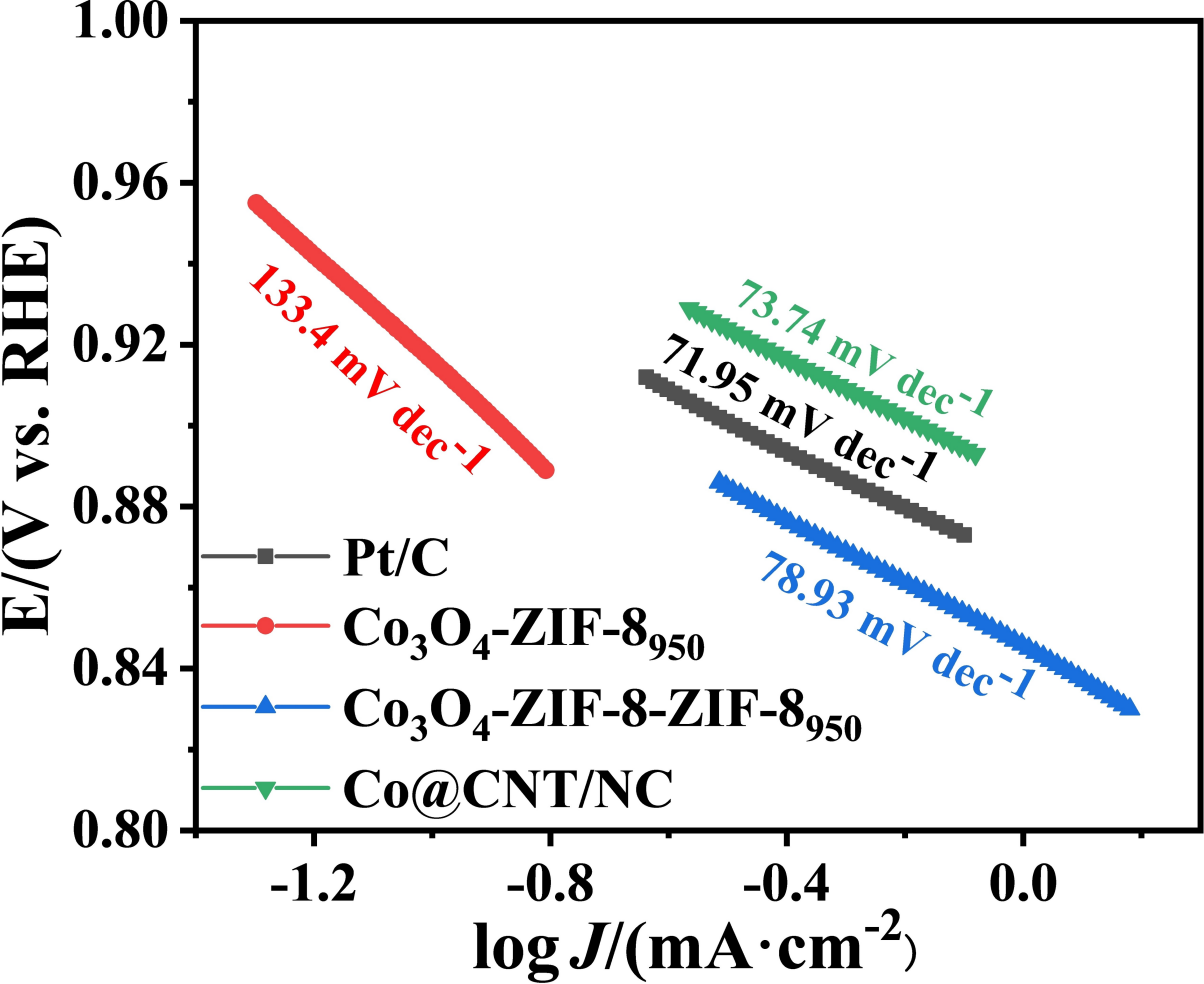
Tafel curves of Pt/C, NC, Co/NC and Co@CNT/NC.

The Tafel curves of Pt, NC, Co/NC and Co@CNT/NC are shown in Figure 8. the Tafel slope of Co@CNT/NC is 73.74 mV dec^−1^, which is slightly higher than that of Pt/C (71.95 mV dec^−1^), but much lower than that of Co/NC and NC. the calculated results of the Tafel slope are consistent with the ORR performance measured by LSV are consistent, indicating that Co@CNT/NC has good ORR reaction kinetics.

In addition, stability is also a key index of catalyst. The ORR durability of Co@CNT/NC was evaluated using chronocurrent method, as shown in Figure S6. After 30000 s cycle, the ORR retention rate of the material is 89.67 %, which is much better than Pt/C, indicating that the material has good ORR stability.

## 2 Conclusions

In this thesis, ZIF‐67‐coated Co_3_O_4_‐decorated ZIF‐8 precursors were prepared by steric encapsulation of ZIFs, and then the carbon nanotube‐coated metal nanoparticle‐decorated polyhedral carbon Co@CNT/NC were prepared by pyrolysis, and the samples showed good ORR and OER electrocatalytic properties due to the high metal modifying and high specific surface area. Meanwhile, to explore further exploration of active sites, direct pyrolysis products of ZIF‐8 (NC) and metal nanoparticles decorated with Co_3_O_4_‐ZIF‐8@ZIF‐8 (Co/NC) were also synthesized as comparison samples with much lower performance than Co@CNT/NC. in addition, a secondary pyrolysis after acid washing was used to remove the carbon nanotubes encapsulated in Co particles and the loaded Co_3_O_4_ nanoparticles. There is a very substantial reduction in their performance, which proves that the carbon nanotubes coated with metal nanoparticles have a great effect on the performance improvement. Therefore, combining ZIF‐67 with ZIF‐8 can retain the metal framework with large specific surface area and porous structure on the one hand, and the carbon tube structure encapsulated with Co nanoparticles and surface‐ modified Co elements can greatly improve the catalytic performance on the other hand. This paper provides a feasible synthetic method for the design of high‐performance electrocatalysts and explores in depth the feasibility of carbon tube‐encapsulated transition metal nanoparticles and metal oxides as catalytic active sites.

## Conflict of Interests

The authors declare no conflict of interest.

## Supporting information

As a service to our authors and readers, this journal provides supporting information supplied by the authors. Such materials are peer reviewed and may be re‐organized for online delivery, but are not copy‐edited or typeset. Technical support issues arising from supporting information (other than missing files) should be addressed to the authors.

Supporting Information
