# Supplementary material for: ZIF‐Co3O4@ZIF‐Derived Urchin‐Like Hierarchically Porous Carbon as Efficient Bifunctional Oxygen Electrocatalysts
Source: ChemistryOpen. 2024 Jun 10;13(9):e202400057. doi: 10.1002/open.202400057 (PMC11467739; doi:10.1002/open.202400057)
Supplement: Supplementary file 1 — Supporting Information [file OPEN-13-e202400057-s001.pdf]

ChemistryOpen

Supporting Information

ZIF-Co_3O_4@ZIF-Derived Urchin-Like Hierarchically Porous Carbon as Efficient Bifunctional Oxygen Electrocatalysts

Lingling Zhang,* Xia Wang, Chong Gong, Weiyan Sun, and Zihan Lu

Supporting Information

ZIF-Co_3O_4@ZIF-Derived Urchin-Like Hierarchically Porous Carbon as Efficient Bifunctional Oxygen Electrocatalysts

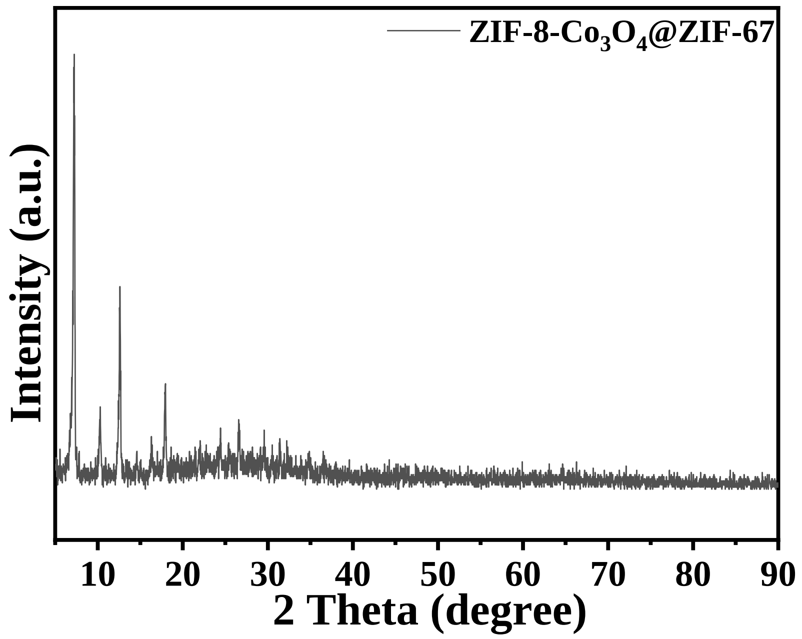

Figure S1. XRD patterns of ZIF-8-Co_3O_4@ZIF-67.

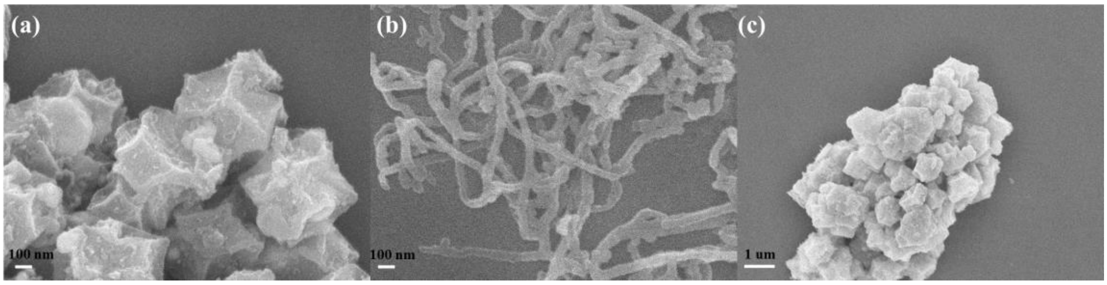

Figure S2. (a) SEM images of pyrolysis products of (a) ZIF-8-Co_3O_4, (b) ZIF-67-Co_3O_4, (c) ZIF-8-Co_3O_4@ZIF-8

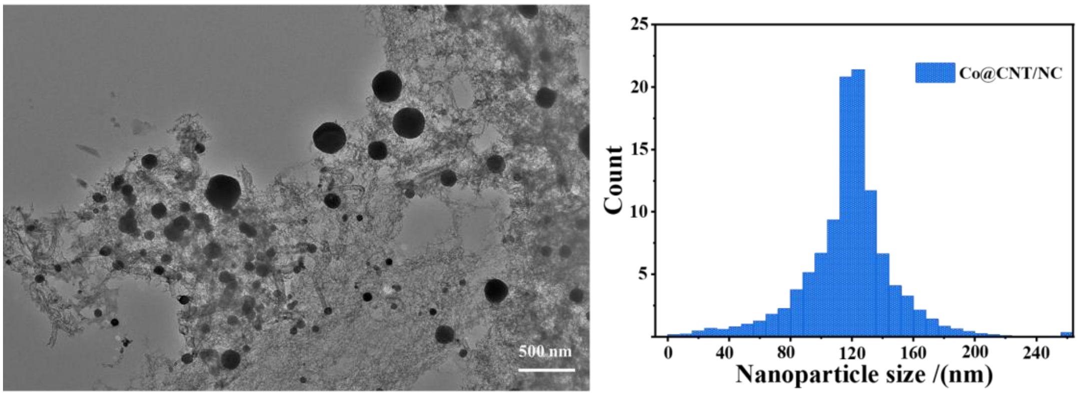

Figure S3. STEM image and Nanoparticle size distribution histogram of Co@CNT/NC.

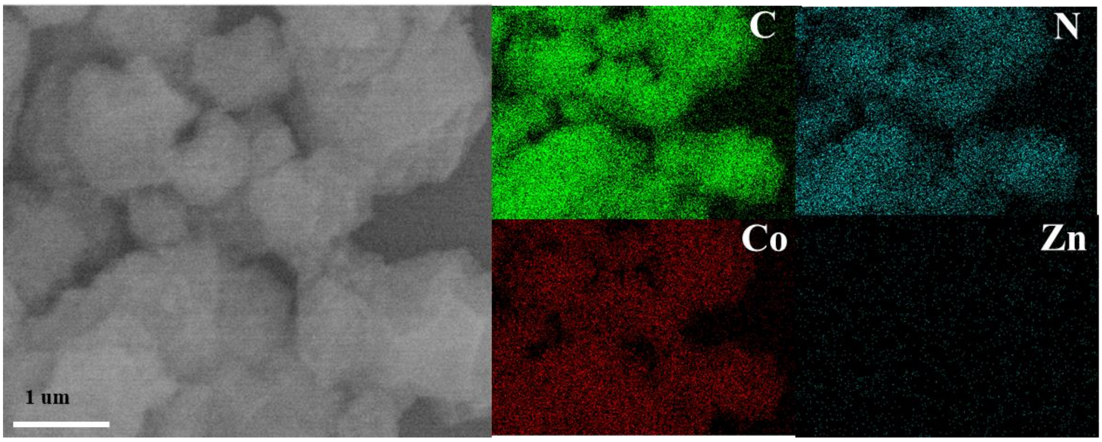

Figure S4. HAADF-STEM image and corresponding elemental mapping images of Co@CNT/NC (C, N, Co, Zn).

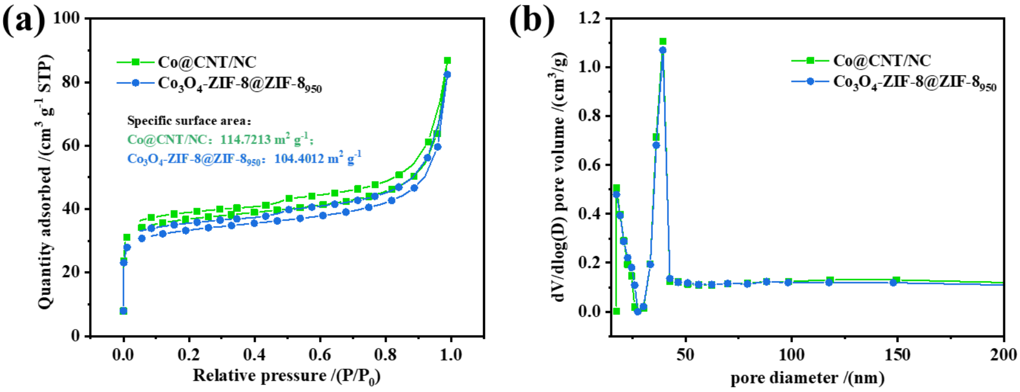

Figure S5. (a) N_2 adsorption/desorption isotherms and (b) corresponding size distributions of mesopores and micropores of Co@CNT/NC and Co_3O_4-ZIF-8@ZIF-8_{950}.

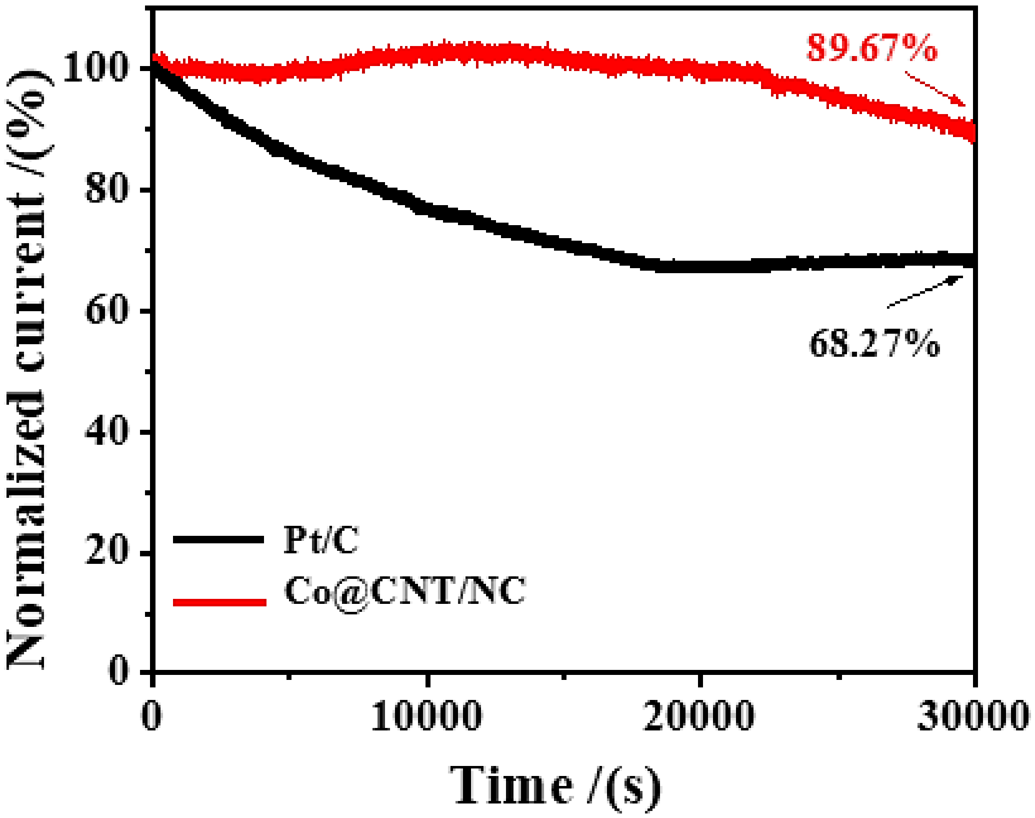

Figure S6. I-t curves of Co@CNT/NC and Pt/C at 0.65 V.

Table S1. Comparison of ORR activities between Co@CNT/NC and noble metal free ORR catalysts reported in recent literature.

Catalyst	$E_{1/2}$ (V vs RHE)	J_l (mA cm^{-2})	$E_{j=10}$ (V vs RHE)	References
Co@NPC/C-MWCNT	0.79	4.39	-	[1]
Co_4/Fe_1@NC	0.83	-	-	[2]
COF@ZIF_{800}	0.85	-	1.38	[3]
FeCo-NPC1100	0.79	5.50	-	[4]
H-NSC@Co/NSC	0.85	-	1.60	[5]
HPCM-5	0.87	6.46	-	[6]
Ce@Co_3O_4/CNFs	0.81	-	1.61	[7]

**Co@CNT/NC**	**0.85**	**4.58**	**1.73**	**This work**

Reference

[1] Z. Liu, D. Ye, X. Zhu, S. Wang, Y. Zou, L. Lan, R. Chen, Y. Yang, Q. Liao, ZIF-67-derived Co nanoparticles embedded in N-doped porous carbon composite interconnected by MWCNTs as highly efficient ORR electrocatalysts for a flexible direct formate fuel cell. Chemical Engineering Journal, 432 (2022) 134192.

[2] A. Han, W. Sun, X. Wan, D. Cai, X. Wang, F. Li, J. Shui, D. Wan, Construction of Co_4 Atomic Clusters to Enable Fe-N_4 Motifs with Highly Active and Durable Oxygen Reduction Performance. Angew Chem Int Ed Engl, 62 (2023) e202303185.

[3] M. Liu, Q. Xu, Q. Miao, S. Yang, P. Wu, G. Liu, J. He, C. Yu, G. Zeng, Atomic Co-N_4 and Co nanoparticles confined in COF@ZIF-67 derived core–shell carbon frameworks: bifunctional non-precious metal catalysts toward the ORR and HER. Journal of Materials Chemistry A, 10 (2022) 228-233.

[4] B. Guo, Q. Ju, R. Ma, Z. Li, Q. Liu, F. Ai, M. Yang, S. Kaskel, J. Luo, T. Zhang, J. Wang, Mechanochemical Synthesis of Multi-Site Electrocatalysts as Bifunctional Zinc-Air Battery Electrodes. Journal of Materials Chemistry A, 7 (2019) 19355–19363.

[5] W. Li, J. Wang, J. Chen, K. Chen, Z. Wen, A. Huang, Core-Shell Carbon-Based Bifunctional Electrocatalysts Derived from COF@MOF Hybrid for Advanced Rechargeable Zn-Air Batteries. Small, 18 (2022) e2202018.

[6] Z. Sun, Y. Wang, L. Zhang, H. Wu, Y. Jin, Y. Li, Y. Shi, T. Zhu, H. Mao, J. Liu, C. Xiao, S. Ding, Simultaneously Realizing Rapid Electron Transfer and Mass Transport in Jellyfish-Like Mott–Schottky Nanoreactors for Oxygen Reduction Reaction. Advanced Functional Materials, 30 (2020).

[7] X. Sun, T. Xu, W. Sun, J. Bai, C. Li, Ce-doped ZIF-67 derived Co_3O_4 nanoparticles supported by carbon nanofibers: A synergistic strategy towards bifunctional oxygen electrocatalysis and Zn-Air batteries. Journal of Alloys and Compounds, 898 (2022).
